# Feasibility of safe outpatient treatment in pediatric patients following intraventricular radioimmunotherapy with ^131^I-omburtamab for leptomeningeal disease

**DOI:** 10.1186/s13550-024-01127-0

**Published:** 2024-07-31

**Authors:** Kavya Prasad, Brian E. Serencsits, Bae P. Chu, Lawrence T. Dauer, Maria Donzelli, Ellen Basu, Kim Kramer, Neeta Pandit-Taskar

**Affiliations:** 1 Department of Medical Physics, Memorial Sloan Kettering Cancer Center, 1275 York Avenue, New York, NY 10065 USA; 2 Department of Pediatrics, Memorial Sloan Kettering Cancer Center, 1275 York Avenue, New York, NY 10065 USA; 3 Molecular Imaging and Therapy Service, Department of Radiology, Memorial Sloan Kettering Cancer Center, 1275 York Avenue, New York, NY 10065 USA

**Keywords:** Brain metastases, Radiopharmaceutical, Radiation safety

## Abstract

**Background:**

Radiolabeled antibody ^131^I-omburtamab was administered intraventricularly in patients with leptomeningeal disease under an institutionally approved study (#*NCT03275402*). Radiation safety precautions were tailored for individual patients, enabling outpatient treatment based on in-depth, evidence-based recommendations for such precautions. The imperative advancement of streamlined therapeutic administration procedures, eliminating the necessity for inpatient isolation and resource-intensive measures, holds pivotal significance. This development bears broader implications for analogous therapies within the pediatric patient demographic.

**Methods:**

Intraventricular radioimmunotherapy (RIT) with 925–1850 MBq (25–50 mCi) of ^131^I-omburtamab was administered via the Ommaya reservoir, in designated rooms within the pediatric ambulatory care center. Dosimeters were provided to staff involved in patient care to evaluate exposure during injection and post-administration. Post-administration exposure rate readings from the patient on contact, at 0.3 m, and at 1 m were taken within the first 30 min, and the room was surveyed after patient discharge. Duration of radiation exposure was calculated using standard U.S. Nuclear Regulatory Commission (US NRC) regulatory guidance recommendations combined with mean exposure rates and whole-body clearance estimates. Exposure rate measurements and clearance data provided patient-specific precautions for four cohorts by age: < 3 y/o, 3–10 y/o, 10–18 y/o, and 18+.

**Results:**

Post-administration exposure rates for patients ranged from 0.16 to 0.46 µSv/hr/MBq at 0.3 m and 0.03–0.08 µSv/hr/MBq at 1 m. Radiation exposure precautions ranged from 1 to 10 days after release for the four evaluated cohorts. Based on the highest measured exposure rates and slowest whole-body clearance, the longest precautions were approximately 78% lower than the regulatory guidance recommendations. Radiation exposure to staff associated with ^131^I-omburtamab per administration was substantially below the annual regulatory threshold for individual exposure monitoring.

**Conclusion:**

^131^I-omburtamab can be administered on an outpatient basis, using appropriate patient-based radiation safety precautions that employ patient-specific exposure rate and biological clearance parameters. This trial is registered with the National Library of Medicine’s ClinicalTrials.gov. The registration number is NCT03275402, and it was registered on 7 September 2017. The web link is included here. https://clinicaltrials.gov/study/NCT03275402.

## Background

Neuroblastoma is a rare form of pediatric cancer of neural crest origin, often metastatic to the central nervous system or the leptomeninges [1, 2]. Intracavitary treatments have been successfully employed, and administration via the Ommaya reservoir treats the central nervous system/leptomeningeal disease [3, 4].

^131^I-omburtamab is a radiolabeled monoclonal antibody (mAbs) that binds to the B7H3 antigen expressed on the tumor cell membrane. Beta emissions of ^131^I, with a maximum energy of 606 keV (abundance of 89%), deliver radiation to the tumor cells, causing DNA damage and cell death [5–8]. While the high-energy, low-range beta particles are the primary source of treatment, the 364 keV gamma ray emitted by ^131^I (81%) is the primary source of radiation exposure to other individuals, including staff, family members, and the general public [8]. The safety profile was determined in a phase 1/2 study of ^131^I-omburtamab (previously 8H9) [1, 9], with doses of 74 MBq (2 mCi) of ^124^I or ^131^I-omburtamab for dosimetry and 370–2960 MBq (10–80 mCi) of ^131^I-omburtamab for therapy, with a recommended phase 2 therapy dose of 1850 MBq (50 mCi) [8, 10–12]. A follow-up multicenter study was conducted with ^131^I-omburtamab in patients with leptomeningeal disease (NCT03275402).

We established and implemented specific radiation safety procedures to enable the administration of intra-Ommaya radioimmunotherapy treatment on an outpatient basis in non-leaded outpatient rooms while maintaining compliance with federal, state, and local regulations. We compared individualized radiation safety precautions, instructions, and parameters based on the recommendations of the U.S. Nuclear Regulatory Commission (US NRC) Regulatory Guide 8.39 to those generated by incorporating exposure rate and clearance data available from monitoring patients following administration of the treatment doses [13].

Here we present the results of the patient-specific instructions provided to parents, caregivers, and potential visitors (or members of the public) and recommendations for combined use of exposure rates and whole-body clearance [14, 15]. We discuss practical and programmatic components for implementing and evaluating exposure and precautions for patients, healthcare providers, and caregivers.

## Methods

### Study design

This manuscript evaluated retrospective data while collecting new information for a prospective study conducted under an approved institutional review board study. The need for informed consent was waived.

### Patients

Exposure rate data from 53 patients, receiving 80 treatments, was analyzed; of these, 12 patients were treated with an activity of 925 MBq (25 mCi) and 1221 MBq (33 mCi) and the remaining were treated with 1850 MBq (50 mCi). An optional dosimetry study evaluated biological clearance data from 28 patients, with a total of 43 treatments. The mean age was 9.8 years (range: 6 months to 18 years).

### Radiopharmaceutical administration room preparation

Patients were treated in MSK’s Pediatric Ambulatory Care Center in preferred rooms in accordance with guidelines for exposure and occupancy. We defined preferred rooms as single-occupancy, corner rooms that shared walls with low-occupancy areas such as stairwells, hallways, and storage rooms, an approach that has been previously described [16]. These rooms were located sufficiently away from public areas such as waiting rooms, play areas, reception desks, and adjacent rooms, with dose rate measurements under 20 µSv in any hour. Before treatment, each room was prepared by radiation safety staff with a waterproof floor covering (polyvinyl chloride PVC), lined trash receptacles, a spill kit, and a radioactive area posting on the door. Following the discharge of the patient, the rooms were surveyed using a Ludlum pancake probe to ensure that contamination levels were below 1000 disintegrations per minute (dpm) over an area of 100 cm^2^, and that all waste was cleared before the room was released back to the unit [16].

### Pre-administration consultation

Health physicists consulted with each patient’s caregivers before treatment, informing them of post-treatment radiation precautions. Instructions were provided regarding maintaining distance, avoiding close contact, and being conscious of bodily fluids. For the relatively younger patient population (< 3 y/o), additional and more involved care was anticipated, including, but not limited to, feeding, changing, and disposal of contaminated diapers and the presence of younger siblings at home. Training was provided to parents and caregivers to assist their child appropriately, detailing care involved in diaper changes, feeding, bathing, and other day-to-day activities while minimizing time spent very close to them, acknowledging that unexpected needs may arise that require more involved care. Electronic dosimeters (Isotrak) were provided to caregivers for the duration of their stay following treatment to study exposure of an individual sitting in the room.

### Dose administration

The radioimmunotherapy dose was administered under aseptic conditions accessed by trained physicians or nurse practitioners in coordination with the nuclear medicine physician and authorized user. A health physicist from the radiation safety service was present to supervise radiation safety aspects during the administration.

### Measurement of radiation exposure

Measurements from the patient were taken within the first 30 min post-injection with a 451B (Fluke Biomedical) ion chamber with the beta window closed for a more accurate energy response to iodine due to the slight over-response for the 364 keV emission with the window open [17]. Measurements were taken on contact with the administration site, at 0.3 m, and at 1 m, in direct line of sight from the injection site or Ommaya reservoir—most often behind the head while the patient was in a supine position, to limit self-shielding and radiation scattering from the patient.

Evaluations for staff and caregiver exposure levels during and post-administration were obtained from single-use electronic dosimeters (DMC 3000 Mirion) worn on the main torso facing the patient to measure deep dose equivalent (DDE) to the whole body. This included the primary registered nurse who cared for the patient for the rest of their stay. Exposure rates for the patient ionization chamber readings and staff dosimeters, measured in milliroentgens per hour (mR/hr). A conversion factor of 1R = 10 mSv was used for all measurements, following the suggestion of the United States Nuclear Regulatory Commission’s Regulatory Guide 8.39 [13]. All results will be further reported in SI units of dose, the micro sievert (µSv). Staff involved in administering the drug were also given two extremity monitors (ring dosimeters Saturn TLD ring (Mirion), one on each hand). Whole body dosimeter data was collected for staff during 16 administrations and extrapolated for the entire population using measured patient ionization chamber exposure rates and mean recorded exposure on the staff dosimeters. This calculation can be seen in Eq. 1 below:

1 $$\:{D}_{N}=\:{D}_{M}\:\:\:\frac{\raisebox{1ex}{${R}_{N}$}\!\left/\:\!\raisebox{-1ex}{${A}_{N}$}\right.}{\raisebox{1ex}{${R}_{M}$}\!\left/\:\!\raisebox{-1ex}{${A}_{M}$}\right.}$$

Where,

D_N_: Extrapolated staff dose, in µSv.

D_M_: Average (mean) staff dose measured by single-use dosimeters, in µSv

R_N_: Dose Rate from a given patient at 1 m, in µSv/hr

A_N_: Radioactivity (I-131) administered to a given patient, in MBq

R_M_: Mean dose rate from ionization chamber readings with staff wearing single-use dosimeters at 1 m, in µSv/hr

A_M_: Mean activity administered to patients with staff using single-use dosimeters, in MBq

Exposure data was further analyzed for different activity levels (925 MBq, 1221 MBq, and 1850 MBq) and corresponding patient age (0–1 y/o, 1–3 y/o, 3–10 y/o, and 10–18 y/o) cohorts to estimate expected exposures to staff and caregivers for the wide range of patients that were treated. The results of these extrapolated values can be seen in Table 2.

### Radiation exposure calculations

Family member/visitor exposures were calculated using modeling based on Release Eq. 2 described by the US NRC in Regulatory Guide 8.39. This calculation can be seen in Eq. 2 below. While this may be a good starting point, Eq. 2 forgoes many patient-specific variables that can more accurately explain an individuals’ release requirements.

2 $$\:D\left(t\right)=\frac{34.6\:\times\:\:\varGamma\:\:\times\:\:{Q}_{O}\times\:\:{T}_{P}\times\:\left(1-{e}^{\frac{-0.693t}{{T}_{P}}}\right)}{{r}^{2}}$$

Where,

D(t) = Accumulated dose at time t, in µSv

34.6 = Conversion factor of 24 h/day times the total integration of decay (1.44)

Γ = Isotope-specific dose rate constant for a point source, µSv/hr*MBq, at 1 cm [18]

Q_0_ = Initial activity of the point source in MBq, at the time of the release

T_E_ = Effective half-life in days

r = Distance from the point source to the point of interest, in centimeters

t = Exposure time in days

$\:\dot{\text{X}}$ = measured dose in µSv/hr at distance r, in cm

$\:\left(1-{e}^{\frac{-0.693t}{{T}_{p}}}\right)$ = radioactive decay factor

To solve this, Eq. 3 was derived to use real-time, patient-based dose rates and biological clearance, as opposed to more generic half-lives and exposure rate constants. Biological clearance data from 28 patients (total of 43 cases) from prior published work was used to calculate an effective half-life ($\:{T}_{E})$ that could be used in the place of physical half-life [14]. Measured, normalized, patient dose rates ($\:\dot{\text{X}}$), in units of $\:\frac{{\upmu\:}\text{S}\text{v}}{\text{h}\text{r}\text{*}\text{M}\text{B}\text{q}}\:$, were substituted for variables $\:\frac{\varGamma\:\:\times\:\:{Q}_{O}}{{r}^{2}}$, with all measurements taken at 1 m. Using measured dose rates allows for further accuracy as it accounts for parameters such as self-shielding and physical distribution in the body, beyond the standard point-source assumption. Calculating the full dose received over the time of decay allows the variable for time (t) to become large and set the radioactive decay factor equal to 1.

3 $$D\left(\infty\right)=\frac{34.6 \times {T}_{E}\times X\times \left(1\right)}{100\,cm^{2}}$$

Regulatory Guide 8.39 allows for the assumption of an occupancy factor of 0.25 at 1 m for patients [13]. This assumption means that an individual will be around the patient for 6 h per day, each day for the lifetime of the radioactivity. For the pediatric population, however, this may not be accurate or conservative enough for real-life scenarios. Instead, occupancy factors specific to varying day-to-day situations can be applied while keeping the per-treatment limit of exposures for caregivers (5 mSv) and members of the public (1 mSv ) in mind [18]. Modified precaution times were modeled on the calculations below where the following situations were evaluated: sleeping apart from other people, including vulnerable populations such as children and pregnant adults, holding a child in one’s lap, and maintaining proper distances from members of the public. An occupancy factor of 0.33, 8 h per day, was used for sleeping precautions at 0.3 m [19]. An additional 0.20 occupancy factor is assumed for holding a child in one’s lap, bathing, feeding, and other close interactions at 0.3 m [19]. An occupancy factor of 0.25 is used for all other interactions at separations of 1 m [19]. If a caregiver or member of the public is expected to be in proximity to a patient for multiple exposure scenarios, such as a parent both holding their child, 0.2 occupancy factor at 0.3 m, and being around them during normal daily activities, 0.25 occupancy factor at 1 m, then the doses received from both scenarios must be added together and equal less than the regulatory limit. Internal institutional recommendations limited the dose received by a caregiver to half of the per-release regulatory dose limit. This was to allow for two treatments to occur, as needed, while still staying below the 5 mSv value. As Low As Reasonably Achievable (ALARA) guidelines can be chosen differently depending on institution preferences.

To calculate the number of days that a patient or caregiver must follow precautions, while still staying below applicable dose limits, a “wait time” for each condition was created. The “wait time” to keep an exposure scenario below a certain dose total, normally 1 or 5 mSv, can be calculated via Eq. 4 [19]. If multiple exposure scenarios are present, their exposures must be added together to stay below applicable limits. The means for splitting the total dose amongst different exposure scenarios is at the discretion of the institution providing the instructions. Results from Eq. 4 will be hereon described as modified precautions, with E(r_j_) in the equation describing the occupancy factor of each scenario [19].

4 $$\:D\left(total\right)=34.6\:\sum\:_{j=1}^{m}E\left({r}_{j}\right)\:\times\:\:\dot{X}({r}_{j},\:0)\:\times\:\:{T}_{E}\times\:{e}^{\frac{-0.693*{t}_{wait}}{{T}_{E}}}$$

The modified precautions calculated using the method described above were presented to the patient and their caregivers pre-administration. Post-administration measurements were collected using a 451 B (Fluke Biomedical) Ion Chamber within the first 30 min and applied to calculations [17]. Parents and caregivers were given instructions based on these modified precautions, which includes patient-specific dose rates and effective half-lives of the pediatric population in questions. As part of this study, these modified instructions were then compared to the instructions created by using the default assumptions in Regulatory Guide 8.39, as seen in Table 3 [13].

The default precautions are represented as Group 1. Three other scenarios for patient release precautions (i.e., longest, median, and shortest) were generated using the normalized exposure rates and biological clearance data. Group 2 comprised the highest exposure scenario, 95th percentile of clearance (highest retention), and 95th percentile of normalized exposure rate. Group 3 was the median exposure scenario—50th percentile of clearance (median retention) and 50th percentile of normalized exposure rates. Group 4 was the lowest exposure scenario: 5th percentile of clearance (lowest retention) and 5% of normalized exposure rates.

## Results

### Radiation exposure and clearance rates

**Fig. 1:**
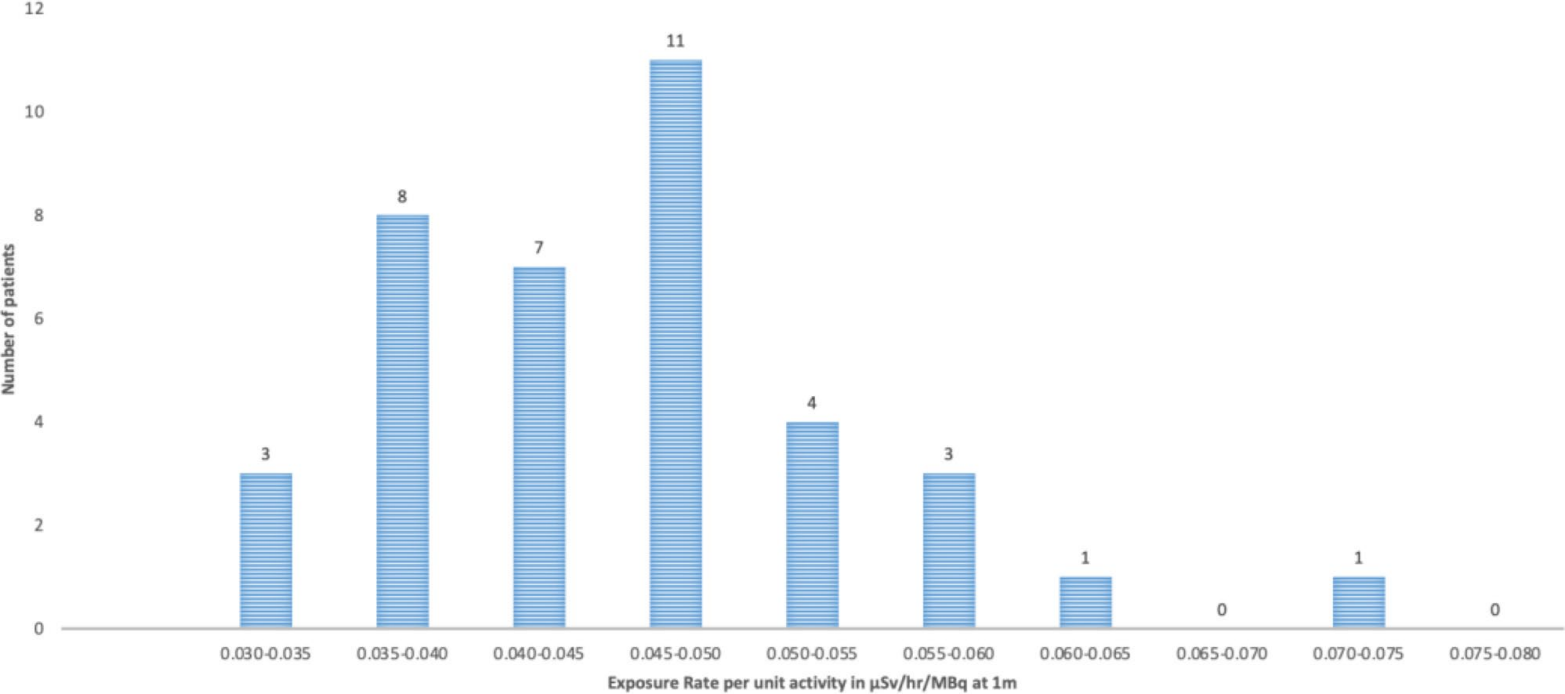
Distribution of normalized exposure rates in µSv/hr/MBq at 1 m

Radiation exposure rate measurements from 53 patients, with a total of 80 treatments, were evaluated. An optional dosimetry study evaluated biological clearance data from 28 patients, with a total of 43 treatments. Median exposure rate measurements were 2100 µSv/hr (1060–7200) on contact, 480 µSv/hr (320–880) at 0.3 m, and 87 µSv/hr (58–148) at 1 m. These measurements were further broken down into four cohorts (Groups A, B, C, and D) and evaluated based on prescribed activity (925, 1221, 1850, and 1850 MBq) and patient age (< 1 y/o, 1–3 y/o, 3–10 y/o, and 10–18 y/o), as described in Table 1. Using the normalized dose measurements, whole-body effective clearance values were calculated to be between 35.9 and 44.2 h, with a range of 23.5 to 69.5 h. The distribution of normalized exposure rates for Groups A-D has also been illustrated in Fig. 1 in units of uSv/hr/MBq at 1 m.

**Table 1:** Distribution of normalized exposure rates in µSv/hr/MBq across the four treatment groups

Group	Prescribed activity	Age	Normalized exposure rate at 0.3 m (µSv/hr/MBq) [median (range)]	Normalized exposure rate at 1 m (µSv/hr/MBq) [median (range)]	Whole-body effective clearance (hrs.) [median (range)]
A	925 MBq	< 1y	0.28 (0.27–0.32)	0.059 (0.054–0.062)	N/A*
B	1221 MBq	1–3 y	0.38 (0.30–0.46)	0.062 (0.051–0.078)	35.9 (33.9–39.8)
C	1850 MBq	3–10 y	0.27 (0.16–0.46)	0.046 (0.032–0.065)	39.7 (25.8–61.0)
D	1850 MBq	10–18 y	0.27 (0.16–0.43)	0.046 (0.030–0.076)	44.2 (23.5–69.5)

*Whole-body effective clearance was not analyzed for this group

### Exposure to caregivers

The median measured exposure to a caregiver obtained over a four-hour post-administration period was 85 µSv, with a range of 50 to 140 µSv, collected for 10 cases.

### Exposure data in staff

The median measured whole-body exposure for the nurse practitioner was 35 µSv, physician was 27 µSv, authorized user was 5 µSv, health physicist was 12 µSv, and registered nurse was 54 µSv. Mean exposure to the staff was evaluated per cohort and recorded as described in Table 2. Most of the exposures on the ring dosimeters resulted in an “M” or minimal (< 100 µSv) value per administration on the report provided by Mirion. Maximum measured extremity exposure was 760 µSv over a 4.5-minute injection time.

**Table 2:** Measured whole-body exposure data per administration from injecting medical doctor (MD)/nurse practitioner (NP), authorized user (AU), registered nurse (RN), and health physicist (HP)

Group*	Activity	Age	Injecting MD/NP (µSv) [median (range)]	Nuclear Medicine AU (µSv) [median (range)]	Primary RN (µSv) [median (range)]	HP (µSv) [median (range)]
B	1221 MBq	1–3 y	30 (26–36)	5 (4–6)	49 (39–59)	15 (12–18)
C	1850 MBq	3–10 y	42 (23–61)	6 (4–9)	69 (39–100)	21 (12–31)
D	1850 MBq	10–18 y	44 (25–67)	7 (4–10)	77 (44–97)	24 (14–30)

*Group A was not evaluated at the time this data was collected

### Algorithm-generated precaution times

The algorithm-generated precaution times are based on the radiation exposure calculations in the methods section using patient-specific exposure rates, occupancy factors, and clearance data. The default precautions are represented as Group 1. Three other scenarios for patient release precautions (i.e., longest, median, and shortest) were generated using the normalized exposure rates and biological clearance data. Group 2 comprised the highest exposure scenario, 95th percentile of clearance (highest retention), and 95th percentile of normalized exposure rate. Group 3 was the median exposure scenario—50th percentile of clearance (median retention) and 50th percentile of normalized exposure rates. Group 4 was the lowest exposure scenario: 5th percentile of clearance (lowest retention) and 5% of normalized exposure rates. The duration of precaution times based on exposure scenarios are evaluated and summarized in Table 3 for a theoretical 1850 MBq dose level patient.

**Table 3:** Duration of precautions in days, following treatment with 1850 MBq, for various scenarios based on exposure and biological retention

Scenario	Group 1	Group 2	Group 3	Group 4
Sleep apart from children and pregnant adults	64	16	7	4
Avoid holding children in lap	59	14	6	3
Sleeping apart from other adults	46	10	4	1
Distance from children, pregnant adults, and members of public	36	8	3	1
Distance from non-pregnant adults	32	7	3	1
Follow Provided Hygiene and Bodily Fluid Precautions	46	10	4	1

## Discussion

The designated treatment rooms are large enough to accommodate the patient, medical equipment, staff, and caregivers while ensuring that exposure measured outside the room is minimal without the use of lead shielding, and following regulations of maintaining dose rates in public areas under 20 µSv/hr, achieved by strategically using rooms further away from public or frequently occupied areas. Using waterproof floor coverings and designated radioactive trash containers allowed all contamination to be properly isolated in the room and subsequently removed for radioactive decay. Radiation safety postings on the door informed staff about precautions, personal protective equipment requirements, and spill response procedures.

The results provided by the electronic whole-body and extremity ring dosimeters demonstrated that staff exposure was minimal, as the duration of close contact was often only a few minutes. Exposure readings of over 100 µSv were attributed to unexpected adverse situations such as vomiting, extensive patient care by the nurse, or spill response. Staff responding to adverse situations donned and doffed personal protective equipment and were surveyed by the health physicist for contamination before exiting the room. The amount of radiation dose received by caregivers during the treatment process, and immediate aftermath, was also found to be minimal. These values did not substantially impact the precautions required for caregivers to remain below the 5 mSv patient-release limit.

Use of biological clearance information introduced an additional factor into exposure calculations that had not been previously employed for assessing exposure and release parameters for pediatric patients receiving radioiodine immunotherapies. Guidance provided in Regulatory Guide 8.39 Revision 1 provides a baseline suggestion for precautions to use physical half-life and no patient self-shielding for patient release purposes. If a licensee considers biological clearance or self-shielding for patient release, these factors must be documented and kept with patient release records. Practically, a patient is a very diffused source of radiation with a large percentage excreted by the body through urine, with trace amounts in sweat and saliva. Effective clearance time was thus much shorter than the physical half-life of the material, resulting in shortened precaution times. Even in the highest exposure scenario (highest exposure rate measurements and retention), the resulting modified precaution times were approximately 78% lower than the default precautions recommended in Regulatory Guide 8.39.

Patients and their families were able to go home after a few hours in the hospital, which allowed them to resume their lives and be secure knowing that they were nearby if they needed medical assistance. The ability to receive treatment and care on an outpatient basis was crucial to the patients’ recovery and response to treatment, as evidenced by improved overall survival through several phases of clinical trials [20]. The benefits of outpatient versus inpatient treatments have been studied extensively, and the advantages of “cancer care outside the hospital walls” can now be safely translated to radioimmunotherapy as well. Extensive education, training, and support must be established with staff prior to implementing such a program in its full scale.

## Conclusion

Our calculations evaluate exposure to caregivers and staff using patient exposure rate measurements and biological clearance data. These calculations allow for continued treatment with ^131^I-omburtamab on an outpatient basis while meeting all regulatory requirements for patient release. Caregivers can stay with their child during and after treatment, with only modest radiation precautions once released from the hospital. Radiation exposure to staff was found to be minimal and was not a limiting factor in outpatient treatment with ^131^I-omburtamab. With proper staff training on basic radiation safety, such as minimizing time spent in very close proximity to the patient, these treatments can be completed in standard treatment rooms without the need for specialized lead-lined rooms, reducing financial burdens and emotional stressors for patients’ families and institutions alike.

## Data Availability

Information regarding this study can be found in the US National Library of Medicine’s Clinical Trial Registry. Web link: https://classic.clinicaltrials.gov/ct2/show/NCT03275402. Additional information is available in a prior published study. Web link: https://jnm.snmjournals.org/content/64/6/946.long. The datasets generated during and/or analyzed during the current study are available from the corresponding author on reasonable request.

## Abbreviations

RIT: Radioimmunotherapy
US NRC: United States Nuclear Regulatory Commission
µSv: Micro sieverts
MBq: Mega becquerel
mAbs: Monoclonal antibodies
MSK: Memorial Sloan Kettering Cancer Center
kEv: Kilo electron volt
mCi: Millicurie
PVC: Polyvinyl chloride
dpm: Disintegrations per minute
y/o: Year(s) old
mSv: Milli sieverts
ALARA: As low as reasonably achievable
